# Concurrent screen use and cross-sectional association with lifestyle behaviours and psychosocial health in adolescent females

**DOI:** 10.1111/apa.15806

**Published:** 2021-03-03

**Authors:** Deirdre M. Harrington, Ekaterini Ioannidou, Melanie J. Davies, Charlotte L. Edwardson, Trish Gorely, Alex V. Rowlands, Lauren B. Sherar, Amanda E. Staiano

**Affiliations:** 1 Diabetes Research Centre, University of Leicester, Leicester, UK; 2 School of Psychological Sciences and Health, University of Strathclyde, Glasgow, UK; 3 Department of Nursing and Midwifery, University of the Highlands and Islands, Inverness, UK; 4 NIHR Leicester Biomedical Research Centre, Leicester, UK; 5 School of Sport, Exercise and Health, Loughborough University, Loughborough, UK; 6 Pennington Biomedical Research Center, Louisiana State University, Baton Rouge, USA

**Keywords:** media, physical activity, sitting positon, sleep, television

## Abstract

**Aim:**

To describe concurrent screen use and any relationships with lifestyle behaviours and psychosocial health.

**Methods:**

Participants wore an accelerometer for seven days to calculate physical activity sleep and sedentary time. Screen ownership and use and psychosocial variables were self-reported. Body mass index (BMI) was measured. Relationships were explored using mixed models accounting for school clustering and confounders.

**Results:**

In 816 adolescent females (age: 12.8 SD 0.8 years; 20.4% non-white European) use of ≥2 screens concurrently was: 59% after school, 65% in evenings, 36% in bed and 68% at weekends. Compared to no screens those using: ≥1 screens at weekends had lower physical activity; ≥2 screens at the weekend or one/two screen at bed had lower weekend moderate-to-vigorous physical activity; one screen in the evening had lower moderate-to-vigorous physical activity in the after-school and evening period; ≥1 screens after school had higher BMI; and ≥3 screens at the weekend had higher weekend sedentary time. Compared to no screens those using: 1–3 after-school screens had shorter weekday sleep; ≥1 screens after-school had lower time in bed.

**Conclusion:**

Screen use is linked to lower physical activity, higher BMI and less sleep. These results can inform screen use guidelines.

## 1 | INTRODUCTION

Screen use is on the rise globally.^1^ Screens are an integral yet concerning part of young people’s lives.^2^ There is interest in the effects of a newer phenomenon where a number of screens are used at the same time. ‘Screen-stacking’, media ‘multi-tasking’, ‘dual screening’ or ‘multi-screen’ viewing/use^3–^^7^ is where, for example, a phone is used at the same time as watching TV and users switch attention rapidly between screens.^7^ This concurrent use of screens was first identified in consumer research in the US (see^7^ for details). Data from time use diaries of 8 to 18 year olds (n = 702) in the US showed an increase in young people who multi-tasked ‘most of the time’ over a five year period.^6^ Data from 1,252 parents/carers of children aged 0–14 in Australia showed that 74% of 10 to 14 year olds do activities on multiple devices.^3^ These data have been supplemented by qualitative data from focus groups with 10 to 11 year olds (n = 63) in the United Kingdom (UK)^4^ and interviews with 11 to 18 year olds and their parents (n = 36) from Singapore ^8^ which showed how multi-tasking came naturally to young people, it was enjoyable and didn’t require much effort.

In the UK there are concerns about the impact of screen use on the mental and physical health of young people, and there have been calls for the development of evidence-based guidance.^9^ Despite this, there is a paucity of conclusive data to support UK evidence-based guidelines on detrimental amounts of screen use.^10^ As there is little evidence about relationships of concurrent screen use to health and wellbeing^11^ the assessment of concurrent screen use is needed to advance the field.^12^ Given the proliferation of screens beyond the TV and mobile phone, data on the prevalence and extent of concurrent viewing that includes a wider range of devices and from multi-ethnic groups would add to the extant literature.

We were interested in understanding the level of concurrent screen use in a cohort of adolescent females in the UK. We also explored whether the use of more screens at the same time was related to less favourable levels of objectively measured lifestyle behaviours and self-reported psychosocial health.

## 2 | METHODS

### 2.1 | Sample

Cross-sectional data (East Midlands, UK; April to June 2016) were used from the final follow-up visit of a cluster randomised controlled trial (RCT) of a school-based physical activity intervention.^13^ As there were no group effects on the primary outcome of moderate-to-vigorous-intensity physical activity (MVPA) at the final (14 month) follow-up ^13^ both groups were combined to conduct a cohort analysis. Participants (secondary school year groups 8 to 10) were randomly selected from all eligible girls in their school at the start of the trial (aged 11 to 14). Questions on screen use were included at the final follow-up data collection only. Participants attending the final follow-up visit (n = 1361) were given these additional questions if they first completed all the main testing procedures (n = 933). Of these participants, those who did not complete the screen use questions were excluded (n = 117) leaving a final analytic sample of 816 participants. Ethics approval was obtained from the University of Leicester ethics sub-committee for Medicine and Biological Sciences. Written informed consent was obtained from the school principal and an opt out consent process was used with parents/guardians. Participants provided verbal assent before completion of any measurement.

### 2.2 | Screen ownership and access

Participants were presented with a list of common screens. Screens were taken from the Screen-Based Media Use Scale (SBMU) ^14^ with other contemporary screens added for the purposes of this study. The original SBMU had test-retest reliability of 0.52.^14^ Participants selected which screens they owned and had access to from a list of nine screens (see Appendix S1).

### 2.3 | Screen usage

Participants self-reported all types of screens they used at the same time ‘*straight after school*’, ‘*evening in your free time*’, ‘*in bed at night*’ and ‘*at weekends*’. Questions from the SBMU were used.^14^ Participants were asked: ‘*Young people sometimes use more than one screen device at the same time (such as a TV on in the background and also browsing on a phone). We call this *‘*multi-screen-viewing*’. *For each of the following times of the day please put a tick next to ALL the screens that you use at the* *same time.*’ Options included nine common screens (see Supplementary file 1). Options of‘*I don’t use any screens at this time* and ‘*Another type of screen*’ were also given.

Concurrent screen use was determined by a simple sum of the number of screens that participants answered that they used at the same time for each time period.

### 2.4 | Dependent variables

Objectively measured physical activity and sedentary time: Participants wore a GENEActiv (Activinsights, Cambs, UK) accelerometer (100 Hz recording) on their non-dominant wrist continuously for seven days.^10^ The.bin files were processed with R-package GGIR version 1.2–2 (http://cran.r-project.org).^15^ The processing steps (including definition of wear time) are included on page 12 of the original study report ^16^ MVPA (≥200 milligravitational (m*g*) units^17^) and average acceleration (Euclidean Norm Minus One or ‘ENMO’), indicative of total volume of PA were calculated. Daytime sedentary time was calculated by subtracting sleep (see below) from total sedentary time. Sedentary time was classified as <40 m*g*.^18^ Variables were calculated for all recorded days and for weekdays, weekend days and for the hours once the participants left school (‘after school+evening’ times based on each school’s end times but approximated 4 to 9 pm).

Objectively measured sleep variables: A number of sleep-related variables were calculated from the GENEActiv data. ‘Time in bed’ represented night time sedentary behaviour which included sleep and any periods awake during the night. ‘Actual sleep duration’ was total time in bed minus the awake periods and essentially is the sleep variable. Both were calculated using the nocturnal sleep detection algorithm in GGIR^19^ and were calculated on all recorded days and for weekdays and weekend days individually. Sleep efficiency was calculated as a measure of the time spent in bed asleep (actual sleep duration/time in bed*100).

Body mass index (BMI): Height (average from two measurements using a Tanita portable stadiometer) and weight (Tanita SC330S bioimpedance scale) were measured to the nearest 0.1 cm and 0.1 kg, respectively. BMI was calculated as weight(kg)/height(m)^2^. BMI z-score was calculated using participant BMI and self-reported age to provide a standardised measure relevant to the UK population.

Self-perceptions: Items from the physical self-perception profile questionnaire assessed self-esteem, physical self-worth and body attractiveness (six statements each).^20^ The original questionnaire included two opposing forced choice statements that were adapted to represent a simple statement that participants responded to on a five point Likert scale from ‘strongly disagree’ to ‘strongly agree’. Negative statements were reversed for analysis.

Health-related quality of life: Participants completed the Child Health Utility-9D, a validated paediatric generic health-related quality of life measure with nine dimensions.^21^ See Supplementary file 1 for the questions.

### 2.5 | Covariates

Self-reported total screen time: Participants completed a modified version of the adolescent sedentary activity questionnaire ^22^ which includes questions on 11 sedentary activities on all days individually. Participants reported how long in hours and minutes they spend in these activities on a typical school day and on a typical weekend day. Only the activities related to leisure time screen use were totalled, that is ‘*watching a programme/film on TV/DVD/tablet/laptop*’, ‘*using the computer/laptop/games console for fun*’ *and* ‘*using a mobile phone/tablet to play games/search the internet/message friends*’. Time spent in these three activities were totalled and split into sample-specific quintiles (for weekdays, Q1: 0.00–3.0 h; Q2: 3.01–5.0 h; Q3: 5.01–6.90 h; Q4: 6.91–10 h; Q5: >10 h; for weekend days, Q1: 0.00–5.0 h; Q2: 5.01–7.0 h; Q3: 7.01–10.0 h; Q4: 10.01–14.23 h; Q5: >14.23 h).

Two independent sample t-tests were performed in order to check differences in the dependent variables between the two ethnic groups (white Europeans and non-white Europeans). Significant differences between the two groups were found in MVPA variables (all-day, after-school, weekdays and weekends), overall PA, all-day and weekday sleep duration, sedentary times (all days, weekdays and weekends) and body attractiveness. In view of this, ethnicity was included as a covariate in all subsequent models.

Other covariates were participant school year group representing age, self-reported ethnicity (white European and non-white European), randomisation arm and accelerometer wear days (wear time). The index of multiple deprivation (IMD) decile (the official measure of relative deprivation of neighbourhoods in England with a lower value meaning more deprived) was calculated^23^ based on participant self-reported postcode.

### 2.6 | Statistical analyses

Participant-level characteristics for the 816 adolescents are presented as means (standard deviation) or numbers (percentages). Missing data for any variable have been identified in the tables and have been removed from the relevant analyses and percentages of the non-missing sample are presented.

Dependent variables were MVPA, BMI z-score, sedentary time, durations of actual sleep and time in bed, self-esteem, self-worth, body attractiveness and health-related quality of life utility index. The dependent variables were checked for normality (via qqplots) and MVPA, sedentary time and weekday and weekend days screen duration deviated slightly. The independent variable of number of screens was collapsed into five (0, 1, 2, 3 and 4+ screens) categories. Linear mixed effects models examined differences in each dependent variable between 1, 2, 3 and 4+ screens versus no screens (0) for each time periods (i.e., after school, in the evening, before bed and at weekends).

All models accounted for school level clustering (school ID; random effects). The models also accounted for covariates listed above. Ethnicity and year group variables were entered as factors (in two and three levels, respectively). Missing data were excluded for all of the variables in each model, and hence, number of observations in each model differs. R-Project for Statistical Computing (v.3.6.1) was used.

## 3 | RESULTS

Participant characteristics are presented in Table 1. Overall, 20.4% of the sample were of non-white European ethnicity, and 26.7% of participants were either overweight or obese. Mean MVPA was 34.4 minutes/day.

### 3.1 | Screen access and usage

Table 2 shows that mobile/smart phones (94.3%) were the most common screen for participants to own (i.e. 79.2% own plus 15.1% own and have access to) followed by tablets (77.3%) and then laptops (71.4%). The proportion of participants reporting the use of each individual screen during each time period is presented in Figure S1. A mobile/smart phone was the most used screen device across the day. A TV was second most used after school, evening and weekend time periods, with a tablet being the second most used before bed.

### 3.2 | Concurrent screen use

Overall, 59%, 65%, 36% and 68% of participants used ≥2 screens concurrently after school, in the evening, before bed and at weekends, respectively. The combination of TV/phone/tablet was the main combination after school (16.5%), evening (24.1%), bed (7.4%) and weekend (36.5%).

### 3.3 | MVPA variables

As seen in Table S1, compared to using no screens, those using one (B = −18.4 mins/day, CI −32.9 to −3.9), two (B = −19.6, CI −33.6 to −5.6), three (B = −22.1, CI −36.0 to −8.2) and four+ (B = −20.9, CI −34.8 to −7.1) screens at weekends had significantly lower MVPA (mins/day) and significantly lower overall PA (1 screen: B = −7.57 m*g*, CI −13.6 to −1.5; 2: B = −8.03, CI −13.9 to −2.2; 3: B = −8.82, CI −14.6 to −3.0; 4+: B = −8.53, CI −14.3 to −2.7). Further, compared to those using no screens, weekend screen use of those using two (B = −23.4 mins/day, CI −43.2 to −3.6), three (B = −25.0, CI −44.7 to −5.4) or four+ (B = −25.1, CI −44.6 to −5.5) screens and one (B = 8.0, CI −14.9 to −1.2) or two (B = −8.6, CI −15.8 to −1.4) bed screens had significantly lower weekend MVPA (mins/day). Those using one screen in the evening had lower (B = −7.1 mins/day, CI-13.6 to −0.5) MVPA in the after-school and evening period.

### 3.4 | Sedentary time

Compared to using no screens, those using two (B = 1.39, CI 0.11 to 2.66), three (B = 1.47 h, CI 0.21 to 2.74) and ≥four (B = 1.38 h, CI 0.12 to 2.64) screens at the weekend had significantly higher weekend sedentary time. No difference was seen between using no screen and one screen (Table S1).

### 3.5 | BMI

Categorical comparisons indicated that compared to using no screens, those using one (B = 0.80 kg/m^2^, CI 0.14 to 1.47), two (B = 0.73 kg/m^2^, CI 0.09 to 1.38), three (B = 0.73 kg/m^2^, CI 0.06 to 1.4) and four+ (B = 0.86 kg/m^2^, CI 0.17 to 1.56) screens after school had significantly higher BMI (Table S2).

### 3.6 | Sleep variables

As seen in Table S2, compared to no screens, those using one (B = −0.44 h, CI −0.87 to −0.01), two (B = −0.54 h, CI −0.95 to −0.12) and three (B = −0.68 h, CI −1.12 to −0.25) after-school screens had significantly lower sleep duration (hours) on weekdays. No difference was seen between 0 and 4 screens. Also, compared to no screens, those using one (B = −0.45 h, CI −0.85 to −0.05), two (B = −0.45 h, CI −0.85 to −0.07), three (B = −0.55 h, CI −0.95 to −0.15) and four+ (B = −0.47 h, CI −0.88 to −0.05) after-school screens had significantly lower time in bed on all days and lower time in bed on weekdays (1 screen: B = −0.54 h, CI −0.99 to −0.08; 2: B = −0.55 h, CI −0.99 to −0.11; 3: B = −0.65 h, CI −1.10 to −0.19; 4+: B = −0.52 h, CI −0.99 to −0.05).

### 3.7 | Psychosocial and health-related quality of life measures

There were no differences in 1,2 or 3 screens versus no screens for any of these self-reported measures. However, compared to using no screens in bed those using 4+ screens had lower self-esteem scores (B = −1.3, CI −2.3 to −0.3).

## 4 | DISCUSSION

Screen use is a prevalent sedentary behaviour that contributes to adolescents’ total sedentary time.^24^ Young people’s engagement in sedentary pursuits has been reported as high with public health concerns about detrimental effects they have on physical and mental wellbeing^25,26^ as well as displacing health behaviours such as physical activity and sleep.

In our analysis, most comparisons between one screen and two or more screens showed no significant relationships. While we queried concurrent use (‘at the same time’) in the time period it is possible that participants were only using one screen at a time most of the time (so the comparisons between 2,3,4+ are actually not comparing anything different which resulted in no significant differences) or that they misinterpreted the question and ticked all the screens they used in a given time period even if not done concurrently. We did find that screen use detrimentally influenced adolescents’ PA (MVPA and overall PA) and sedentary time on weekends, sleep time and also they had higher BMI.

Until recently the majority of screen use was via traditional TV,^24^ but contemporary adolescents have access to and use a wider variety of screens. In a large multi-ethnic UK sample, more than 70% of young adolescent females used at least one screen within the hour before bed with phones, tablets and TV also being the most commonly used devices.^27^Much of the evidence on the associations of screens with adolescents’ health focus on screen use via traditional TV viewing.^28^ To take into account the variety of screens now available to young people, recent studies such as ours have assessed ‘new modes’ of TV viewing ^29^ or engagement with other screens such as mobile phones, personal computers and tablets.

The magnitude of the effect of screen use in our analysis is substantial. For example, a participant using any number of screens at the weekend would have approximately 20 minutes lower MVPA per day and approximately 8 *mg* lower mean acceleration per day than a participant using no screens at the weekend. Just using a screen is the issue here rather than the fact there are multiple screens used at once. We found that a participant using two or more screens concurrently at the weekend would have approximately 1 hour 25 minutes higher sedentary time per day at the weekend and approximately 24 minutes lower MVPA per day at the weekend compared to those using no screen. Less favourable sedentary and MVPA levels were not seen for those using one screen only at the weekend.

We found that participants using any number of screens concurrently after school would have a BMI 0.80 units higher than someone using no screens after school. Similarly, those using one, two or three screens concurrently after school would have approximately 30 minutes lower sleep duration than someone using no screens after schools. A possible reason for why the after-school period is critical could be that adolescents use their screen(s) after school to ‘catch up’ on their screen-based media activity, then move to other daily tasks like homework, chores and family time which subsequently encroaches into sleeping time.

Overall our results indicate that the use of a screen rather than concurrent screen use that is detrimental except in the case of the relationships between weekend screen use. Higher weekend sedentary time was seen for the use of two (1 h 23 mins), three (1 h 28 mins) or four screens (1 h 22 mins) but not one (1 h; NS) compared to no screen use.

We hypothesised that more screens used concurrently would be associated with less favourable levels of psychosocial measures. Other than those using four or more screens in bed having lower self-esteem scores we did not find any associations with self-worth, self-rated body attractiveness or health-related quality of life. Data on the relationships between concurrent screen use and mental health-related measures are not well known. Although evidence from a large Australian sample of children and young adolescents linking screen time to poorer mental health scores in adolescents,^30^ there is still little compelling evidence of a link between screen use and negative psychological wellbeing including self-esteem.^31^ The evidence base is such that the UK Chief Medical Officers deemed creating guidance premature.^10^ Screen use guidelines need to be evidence-based and our data adds to the evidence that may shift the focus to how excessive screen use certain times of the day can affect young people’s lifestyle behaviours.

The limitations of this analysis include the all-female sample from one geographic location of the UK. Data from the United States have reported higher levels of multi-tasking in girls^6^ so it would be reasonable to assume a sample including boys may yield different results. There are also the accepted limitations of wrist-based accelerometry to assess sedentary time. It is possible that the question on screens used ‘at the same time’ was interpreted as all the screens used in the time period even if not used concurrently. This is particularly evident for the ‘in bed at night’ period where a small number of participants reported using four screens concurrently. This means the result linking four or more screens to self-esteem should be interpreted with caution. Strengths include the multi-ethnic sample of adolescents with excellent compliance to the accelerometer protocol allowing objective assessments of sedentary time and sleep which is a unique contribution to the literature. The number of significant findings must be held relative to the number of null findings.

In summary, the prevalence of concurrent screen use is high in adolescents and screen use is linked to lower PA, higher BMI and less sleep in this cohort. In our analysis the use of multiple screens did not appear to be more detrimental than screen use itself. However, this could be due to the interpretation of the question by participants. Concurrent screen use may pose an opportunity to target young people with interventions or health-related content through multiple devices. This study adds to the evidence base to inform future screen use guidelines.

## Supplementary Material

Figure S1

Tables S1-S2

Appendix S1

## Figures and Tables

**TABLE 1:** Participant characteristics (n = 816)

Individual level characteristics	Total (n = 816)
Age, years (SD)	12.8 (0.8)

Year group categories, n (%)	
Year 7	329 (40.3)
Year 8	314 (38.5)
Year 9	173 (21.2)

Ethnicity categories, n (%)	
White European	647 (79.6)
Non-white European	166 (20.4)

IMD, decile (SD)	5.74 (2.8)

IMD quintiles, n (%)	
Q1	124 (15.5)
Q2	136 (16.7)
Q3	152 (18.6)
Q4	189 (23.2)
Q5	155 (19.0)

Intervention group, n (%)	633 (77.6)

BMI, z-score (SD)	0.15 (1.34)

BMI category, n (%)	
Underweight	44 (5.4)
Normal weight	543 (66.5)
Overweight	145 (17.8)
Obese	73 (8.9)

*Accelerometer variables*, mean (SD)	
MVPA, mins/day^a^	34.4 (8.5)
Light PA, mins/day	275.6 (44.6)
Sedentary, mins/day^a^	1127.0 (62.0)
Overall PA, *mg*^a^	43.0 (20.5)
Average wear days, valid days	6.34 (1.38)
Actual sleep duration, hours/24-h^a^	7.7 (0.84)

*Psychosocial variables*, mean (SD)	
Self-esteem, score	17.8 (2.4)
Self-worth, score	17.6 (2.7)
Body attractiveness, score^a^	16.6 (3.0)
Utility score for health-related quality of life, score^¶^	0.8 (0.1)

BMI, body mass index; IMD, participant index of multiple deprivation (a measure of socio-economic status with lower values indicating higher deprivation); m*g*, milligravitational units; MPVA, moderate-to vigorous-intensity physical activity; SD, standard deviation. Missing data: IMD (n = 60), BMI (n = 11), accelerometer variables (n = 170), light PA (n = 10), ethnicity (n = 3), self-esteem, self-worth, body attractiveness (n = 183), utility score (n = 154). Psychosocial variables had a maximum score of 30

¶ health-related quality of life score has a maximum score of 1 which is ‘perfect health’.

a Differences by ethnicity grouping (white Europeans and non-white Europeans).

**TABLE 2:** Access to common screen-based media

	Gaming system	TV	Mobile or smart phone	Laptop/ notebook/ netbook/ Mac	Tablet	PC	PlayStation Portable/ Nintendo DS	iPod Touch/ touch screen music player	eReader
Owns	258 (40.7)	349 (55.0)	502 (79.2)	373 (58.8)	403 (63.6)	170 (26.8)	292 (46.1)	317 (50.0)	150 (23.7)
Has access to	237 (37.4)	157 (24.8)	27 (4.3)	149 (23.5)	115 (18.1)	283 (44.6)	186 (29.3)	144 (22.7)	221 (34.9)
Owns and has access to	77 (12.1)	92 (14.5)	96 (15.1)	80 (12.6)	87 (13.7)	44 (6.9)	62 (9.8)	61 (9.6)	40 (6.3)
Neither	59 (9.3)	33 (5.2)	6 (0.9)	29 (4.6)	26 (4.1)	134 (21.1)	91 (14.4)	109 (17.2)	220 (34.7)

N = 631; 185 of the 816 did not answer this question; values presented are number in each group with percentage of sample in parenthesis.

## Data Availability

Owing to the use of opt out consent, and not including any specific data sharing information in the participant and parent/guardians information sheets, there are no data that can be shared publicly. Please contact the corresponding author for further details.

## Key points

- Concurrent screen use was prevalent across the day in this sample of females adolescents in the UK.
- Two or more screens were used concurrently by 59% of adolescents after school, 65% in the evenings, 36% in bed and 68% at weekends.
- Screen use, rather than number of screens used at the same time, was related to lower physical activity, higher BMI and less sleep in adolescents.

## Abbreviations:

BMI: Body mass index
ENMO: Euclidean norm minus one
IMD: Index of multiple deprivation
MVPA: Moderate-to-vigorous-intensity physical activity
PA: Physical activity
RCT: Randomised controlled trial
SBMU: Screen-Based Media Use Scale
UK: United Kingdom
