# Supplementary figures and images for: Concurrent screen use and cross-sectional association with lifestyle behaviours and psychosocial health in adolescent females
Source: Acta Paediatr. Author manuscript; Available in PMC 2022 May 26. (PMC9134851; doi:10.1111/apa.15806)

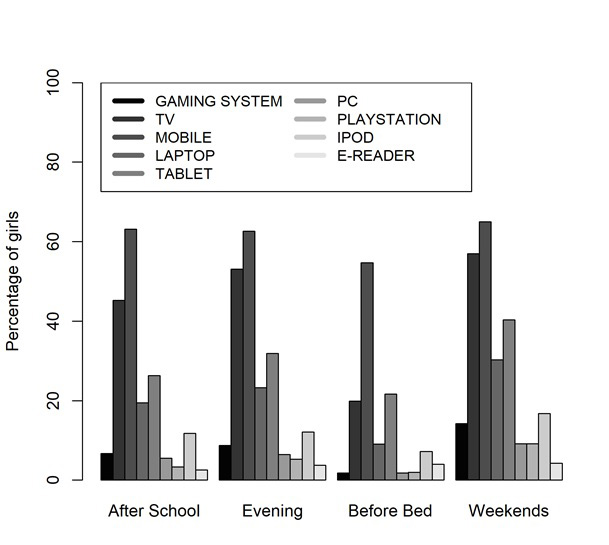

Supplement: Figure S1 [file NIHMS1802740-supplement-Figure_S1.jpg]
