# Supplementary material for: Concurrent screen use and cross-sectional association with lifestyle behaviours and psychosocial health in adolescent females
Source: Acta Paediatr. Author manuscript; Available in PMC 2022 May 26. (PMC9134851; doi:10.1111/apa.15806)
Supplement: Tables S1-S2 [file NIHMS1802740-supplement-Tables_S1-S2.docx]

**Table S1.** Relationship between number of screens viewed concurrently with MVPA and sedentary time variables

|  | **All days MVPA (min/day)** | | | | | **After school + evening MVPA (min/day)** | | | | **School day MVPA (min/day)** | | | | | **Weekend MVPA (min/day)** | | | | |
| --- | --- | --- | --- | --- | --- | --- | --- | --- | --- | --- | --- | --- | --- | --- | --- | --- | --- | --- | --- |
|  | After school | Evening | Bed | Weekend | After school | | Evening | Bed | Week end | After school | Evening | Bed | Week end | After school | | Evening | Bed | Weekend |  |
| **0** | ref | ref | ref | ref | ref | | ref | ref | ref | ref | ref | ref | ref | ref | | ref | ref | ref |  |
| **1** | 0.94  (-9.53, 11.42) | -7.47  (-19.18, 4.24) | -3.70  (-8.75, 1.36) | **-18.37**  **(-32.87, -3.88)** | -1.63  (-7.50, 4.24) | | **-7.05**  **(-13.58, -0.52)** | - | - | 0.82  (-10.07, 11.72) | -8.83  (-21.00, 3.33) | -2.33  (-7.59, 2.93) | - | - | | -4.25  (-19.85, 11.34) | **-8.03**  **(-14.89, -1.17)** | -19.76  (-40.26, 0.75) |  |
| **2** | -1.09  (-11.22. 9.04) | -6.50  (-17.71, 4.71) | -4.84  (-10.13, 0.45) | **-19.57**  **(-33.57, -5.57)** | -2.43  (-8.10, 3.25) | | -6.03  (-12.28, 0.22) | - | - | -0.76  (-11.30, 9.78) | -6.40  (-18.05, 5.24) | -4.11  (-9.62, 1.40) | - | - | | -7.36  (-22.23, 7.52) | **-8.56**  **(-15.78, -1.35)** | **-23.41**  **(-43.17, -3.64)** |  |
| **3** | 0.88  (-9.63, 11.39) | -6.64  (-17.94, 4.66) | -4.44  (-11.82, 2.94) | **-22.08**  **(-36.00, -8.17)** | -1.99  (-7.88, 3.90) | | -6.14  (-12.44, 0.16) | - | - | 1.11  (-9.82, 12.04) | -8.05  (-19.79. 3.68) | -3.79  (-11.47, 3.89) | - | - | | -5.34  (-20.36, 9.69) | -8.27 (-18.48, 1.93) | **-25.03**  **(-44.68, -5.38)** |  |
| **4** | 0.77  (-10.19, 11.73) | -5.83  (-17.26, 5.59) | 0.21  (-7.78, 8.20) | **-20.94**  **(-34.77, -7.11)** | -2,27  (8.41, 3.87) | | -4.77  (-11.14, 1.60) | - | - | 0.64  (-10.76, 12.04) | -6.56  (-18.43, 5.31) | 0.86  (-7.46, 9.17) | - | - | | -4.98  (-20.15, 10.20) | -1.21  (-12.08, 9.66) | **-25.05**  **(-44.58, -5.52)** |  |

**Table S1. cont.**

|  | **ENMO (m*g)*** | | | | **All days sedentary time (hrs/day)** | | | | | **School day sedentary time (hrs/day)** | | | | | **Weekend sedentary time (hrs/day)** | | | |
| --- | --- | --- | --- | --- | --- | --- | --- | --- | --- | --- | --- | --- | --- | --- | --- | --- | --- | --- |
|  | After school | Evening | Bed | Weekend | After school | Evening | Bed | Weekend | After school | | Evening | Bed | Week end | After school | | Evening | Bed | Weekend |
| **0** | ref | ref | ref | ref | ref | ref | ref | ref | ref | | ref | ref | ref | ref | | ref | ref | ref |
| **1** | -0.37  (-4.72, 3.98) | -2.16  (-7.03, 2.71) | -1.48  (-3.58, 0.62) | **-7.57**  **(-13.64, -1.50)** | 0.33  (-0.30, 0.96) | -0.10  (-0.81, 0.61) | 0.22  (-0.084,0.53) | 0.48  (-0.39, 1.35) | 0.44  (-0.20, 1.08) | | 0.20  (-0.52, 0.91) | 0.23  (-0.08, 0.54) | - | - | | -0.62  (-1.66, 0.42) | 0.33  (-0.11, 0.78) | 1.05  (-0.28, 2.37) |
| **2** | -0.67  (-4.88, 3.54) | -1.68  (-6.34, 2.98) | -1.65  (-3.85, 0.55) | **-8.03**  **(-13.90, -2.17)** | 0.31  (-0.31, 0.92) | -0.03  (-0.71, 0.65) | 0.20  (-0.13, 0.52) | 0.68  (-0.16, 1.52) | 0.44  (-0.17, 1.06) | | 0.15  (-0.53, 0.84) | 0.21  (-0.12, 0.54) | - | - | | -0.42  (-1.42, 0.57) | 0.25  (-0.22, 0.72) | **1.39**  **(0.11, 2.66)** |
| **3** | 0.14  (-4.22, 4.51) | -1.11  (-5.81, 3.58) | -2.16  (-5.23, 0.91) | **-8.82**  **(-14.65, -2.99)** | 0.36  (-0.28, 0.99) | 0.0004  (-0.68, 0.68) | 0.31  (-0.14, 0.76) | 0.88  (0.05, 1.72) | 0.53  (-0.11, 1.18) | | 0.25  (-0.44, 0.94) | 0.30  (-0.16, 0.75) | - | - | | -0.48  (-1.49, 0.52) | 0.60  (-0.06, 1.26) | **1.47**  **(0.21, 2.74)** |
| **4** | 0.50  (-4.06, 5.05) | -1.14  (-5.89, 3.61) | 0.16  (-3.16, 3.48) | **-8.53**  **(-14.32, -2.73)** | 0.08  (-0.58, 0.74) | -0.20  (-0.89, 0.49) | -0.16  (-0.65, 0.32) | 0.64  (-0.19, 1.47) | 0.14  (-0.53, 0.81) | | -0.11  (-0.81. 0.59) | -0.13  (-0.63, 0.36) | - | - | | -0.31  (-1.32, 0.70) | -0.21  (-0.92, 0.50) | **1.38**  **(0.12, 2.64)** |

**Note:** values shown are *B* coefficients with confidence internals in parenthesis

**Table S2.** Relationship between number of screens viewed concurrently with BMI and sleep variables

|  | **BMI z-score** | | | | **Actual sleep duration all days (hrs/day)** | | | | | **Actual sleep duration weekdays (hrs/day)** | | | | | **Actual sleep duration weekends (hrs/day)** | | | | |
| --- | --- | --- | --- | --- | --- | --- | --- | --- | --- | --- | --- | --- | --- | --- | --- | --- | --- | --- | --- |
|  | After school | Evening | Bed | Weekend | After school | Evening | Bed | Weekend | After school | | Evening | Bed | Week end | After school | | Evening | Bed | Weekend |  |
| **0** | ref | ref | ref | ref | ref | ref | ref | ref | ref | | ref | ref | N/A | N/A | | ref | ref | ref |  |
| **1** | **0.80**  **(0.14, 1.47)** | -0.16  (-0.91, 0.59) | 0.01  (-0.31, 0.34) | -0.22  (-1.17, 0.73) | -0.30  (-0.71, 0.10) | 0.12  (-0.33, 0.57) | -0.009  (-0.21, 0.19) | 0.26  (-0.29, 0.82) | **-0.44**  **(-0.87, -0.01)** | | -0.11  (-0.59, 0.38) | -0.10  (-0.31, 0.11) | - | - | | 0.66  (0.01, 1.31) | 0.16  (-0.12, 0.44) | -0.37  (-1.20, 0.47) |  |
| **2** | **0.73**  **(0.09, 1.38)** | -0.19  (-0.91, 0.53) | 0.04  (-0.30, 0.38) | -0.14  (-1.06, 0.78 ) | -0.35  (-0.74, 0.04) | -0.08  (-0.51, 0.35) | -0.08  (-0.29, 0.13) | 0.044  (-0.50, 0.59) | **-0.54**  **(-0.95, -0.12)** | | -0.23  (-0.69, 0.23) | -0.11  (-0.33, 0.12) | - | - | | 0.47  (-0.15, 1.09) | 0.09  (-0.21, 0.39) | -0.48  (-1.28, 0.33) |  |
| **3** | **0.73**  **(0.06, 1.40)** | -0.20  (-0.92, 0.53) | 0.06  (-0.42, 0.53) | -0.27  (-1.18, 0.65) | -0.47  (-0.88, -0.07) | -0.19  (-0.63, 0.24) | -0.01  (-0.30, 0.28) | -0.07  (-0.61, 0.47) | **-0.68**  **(-1.12, -0.25)** | | -0.37  (-0.83, 0.10) | -0.006  (-0.31, 0.30) | - | - | | 0.42  (-0.20, 1.05) | 0.03  (-0.37, 0.44) | -0.48  (-1.28, 0.32) |  |
| **4** | **0.86**  **(0.17, 1.56)** | -0.06  (-0.80, 0.67) | 0.34  (-0.17, 0.86) | -0.04  (-0.95, 0.86) | -0.36  (-0.78, 0.06) | -0.04  (-0.48, 0.39) | -0.0005  (-0.31, 0.31) | 0.13  (-0.41, 0.66) | -0.45  (-0.90, 0.0009) | | -0.12 (-0.59, 0.35) | -0.007  (-0.34, 0.33) | - | - | | 0.26  (-0.37, 0.90) | -0.002  (-0.45, 0.44) | -0.51  (-1.30, 0.29) |  |

**Table S2. cont.**

|  | **Time in bed duration all days (hrs/day)** | | | | | **Time in bed duration weekdays (hrs/day)** | | | | | **Time in bed duration weekends (hrs/day)** | | | | | **Sleep efficiency (%)** | | | |
| --- | --- | --- | --- | --- | --- | --- | --- | --- | --- | --- | --- | --- | --- | --- | --- | --- | --- | --- | --- |
|  | After- school | Evening | Bed | Weekend | After- school | | Evening | Bed | Week end | After- school | | Evening | Bed | Weekend | After- school | | Evening | Bed | Weekend |
| **0** | ref | ref | ref | ref | ref | | ref | ref | N/A | N/A | | ref | ref | ref | ref | | ref | ref | ref |
| **1** | **-0.45**  **(-0.85, -0.05)** | 0.23  (-0.21, 0.68) | 0.22  (-0.02, 0.41) | 0.36  (-0.20, 0.92) | **-0.54**  **(-0.99, -0.08)** | | 0.01  (-0.49, 0.52) | 0.16  (-0.06, 0.38) | - | - | | 0.71  (0.009, 1.42) | 0.32  (0.01, 0.62) | 0.06  (-0.85, 0.96) | 0.003  (-0.03, 0.04) | | -0.006  (-0.04, 0.03) | -0.02  (-0.03, -0.003) | -0.008  (-0.05, 0.04) |
| **2** | **-0.45**  **(-0.84, -0.07)** | 0.03  (-0.40, 0.46) | 0.10  (-0.11, 0.30) | 0.23  (-0.31, 0.77) | **-0.55**  **(-0.99, -0.11)** | | -0.19  (-0.67, 0.30) | 0.07  (-0.16, 0.30) | - | - | | 0.58  (-0.10, 1.26) | 0.27  (-0.05, 0.59) | 0.01  (-0.86, 0.89) | -0.0002  (-0.03, 0.03) | | -0.010  (-0.04, 0.02) | -0.02  (-0.03, 0.001) | -0.02  (-0.06, 0.02) |
| **3** | **-0.55**  **(-0.95, -0.15)** | -0.08  (-0.51, 0.35) | 0.10  (-0.19, 0.38) | 0.12  (-0.42, 0.66) | **-0.65**  **(-1.10, -0.19)** | | -0.31  (-0.80, 0.17) | 0.18  (-0.14, 0.50) | - | - | | 0.61  (-0.07, 1.29) | 0.02  (-0.42, 0.46) | 0.02  (-0.85, 0.89) | -0.004  (-0.04, 0.03) | | -0.010  (-0.04, 0.03) | -0.008  (-0.03, 0.01) | -0.02  (-0.07, 0.02) |
| **4** | **-0.47**  **(-0.88, -0.05)** | 0.11  (-0.32, 0.55) | 0.18  (-0.13, 0.49) | 0.28  (-0.25, 0.81) | **-0.52**  **(-0.99, -0.05)** | | -0.05  (-0.54, 0.44) | 0.20  (-0.15, 0.55) | - | - | | 0.58  (-0.11, 1.26) | 0.17  (-0.31, 0.65) | -0.07  (-0.93, 0.80) | -0.002  (-0.04, 0.03) | | -0.01  (-0.05, 0.02) | -0.02  (-0.04, 0.009) | -0.02  (-0.06, 0.03) |

**Note:** values shown are *B* coefficients with confidence internals in parenthesis
