# Supplementary material for: Concurrent screen use and cross-sectional association with lifestyle behaviours and psychosocial health in adolescent females
Source: Acta Paediatr. Author manuscript; Available in PMC 2022 May 26. (PMC9134851; doi:10.1111/apa.15806)
Supplement: Appendix S1 [file NIHMS1802740-supplement-Appendix_S1.docx]

**Appendix S1**

Screen ownership and access

Participants ticked which screens they owned and which they had access to. The options were*:* Gaming system (e.g. Xbox^TM^, PlayStation^TM^, GameCube^TM^, Wii^TM^, Kinect^TM^); TV; Mobile or smart phone (iPhone ^TM^, Android phone, etc.); Laptop/ notebook/ netbook/ Mac ^TM^; Tablet (iPad^TM^, Android^TM^, etc.); PC (desktop computer); PlayStation Portable^TM^ / Nintendo DS^TM^; iPod ^TM^ Touch/ touch screen music player; and eReader (e.g. Kindle^TM^, Nook^TM^).

Screen usage

The nine common screens included were: iPod^TM^ Touch/touch screen music player; iPad ^TM^, android tablet or other tablet; iPhone^TM^, Android ^TM^ phone or other mobile phone; TV (watching); TV (used for gaming); Laptop/notebook/netbook/Mac ^TM^; PC (desktop computer); PlayStation Portable^TM^ /Nintendo DS^TM^; eReader (e.g. Kindle^TM^, Nook^TM^)*.* Options of “I don’t use any screens at this time” and “Another type of screen” were also given.

Health related quality of life

For five dimensions (worried, sad, pain, tired, annoyed), participant responses could range from “I don’t feel [given dimension] today” to “I feel very [given dimension] today.” For ability to do school work/homework and their daily routine, participant responses could range from “I have no problems with [given dimension] today” to “I can’t do [given dimension] today.” For sleep and daily activities participants could respond with “Last night I had no problem sleeping” to “Last night I couldn’t sleep at all” and “I can join in with any activities today” to “I can join in with no activities today”.
